# Influence of Habitat Alteration on the Molecular Profile of Membrane Lipids of the Coral *Junceella fragilis*

**DOI:** 10.3390/biology15080602

**Published:** 2026-04-10

**Authors:** Elena T. Bizikashvili, Tatyana V. Sikorskaya, Kseniya V. Efimova, Ekaterina V. Ermolenko

**Affiliations:** A.V. Zhirmunsky National Scientific Center of Marine Biology, Far Eastern Branch, Russian Academy of Sciences, ul. Palchevskogo 17, 690041 Vladivostok, Russia; miss.tatyanna@yandex.ru (T.V.S.); xengen88@gmail.com (K.V.E.); ecrire_711@mail.ru (E.V.E.)

**Keywords:** HPLC-MS/MS, gorgonian, *Symbiodinium*, lipidomics, genetic analysis, membranes

## Abstract

The cell membrane serves as the first line of defense against adverse environmental factors and is first to adapt to changing conditions. Cell membranes in both coral and its symbionts, which use different membrane adaptation strategies, have to acclimatize to various abiotic stressors. As our molecular-genetics analysis showed, colonies of *Junceella fragilis* were associated with dinoflagellates *Cladocopium thermophilum, Gerakladium endoclionum* and *Breviolum minutum*. We analyzed the phospholipid (PL) molecular species of the wild and cultivated *Junceella fragilis* and their dinoflagellates (phosphatidylcholine (PC), phosphatidylethanolamine (PE), phosphatidylserine (PS), phosphatidylinositol (PI), ceramideaminoethylphosphonate (CAEP)), as well as thylakoid membrane lipids of dinoflagellates (glycolipids and betaine lipids). When comparing wild and cultivated *J. fragilis* colonies, there were no significant differences in thylakoid lipids, but there were differences in host membrane phospholipids, namely in PC, PE and PS. Thus, the profile of PL molecular species of the membrane is very sensitive to environmental factors, which probably explains the observed differences in the profiles of molecular PL species in this study.

## Simple Summary

This study presents a comparative lipidomic and molecular genetics analysis of the coral *Junceella fragilis* and its endosymbionts from the family Symbiodiniaceae. Specimens collected from the wild (South China Sea) were compared with colonies acclimatized for one year under controlled aquarium conditions. The genetic analysis confirmed the stability of the symbiotic community: the same dominant symbiont species (*Cladocopium thermophilum* and *Gerakladium endoclionum*) were identified in both wild and cultivated colonies. As shown by the lipidomic analysis using HPLC-MS/MS, the composition of the symbionts’ thylakoid lipids (glycolipids and betaine lipids), as well as the host’s phosphatidylinositols (PI) and ceramideaminoethylphosphonates (CAEP), did not change significantly. Key differences were found in the molecular species of phospholipids (PL) comprising the host plasma membrane, primarily ether-linked forms of phosphatidylcholine (PC), phosphatidylethanolamine (PE), and phosphatidylserine (PS). The increased content of ether-linked PS and PE in cultivated colonies may indicate membrane adaptation to variations in abiotic factors (e.g., light intensity) by enhancing their antioxidant potential and stability. The plasma membrane of the coral host exhibited greater lipid compositional plasticity in response to changes in light intensity and temperature under aquarium conditions, while the symbiont’s lipidome and host’s lipid classes (PI, CAEP) remained conserved.

## 1. Introduction

As members of the phylum Cnidaria, anthozoan corals (Anthozoa) are ranked among the most vital marine organisms for their role in constructing reef ecosystems. The class Anthozoa is divided into two subclasses: Hexacorallia and Octocorallia. Scleractinian corals (Anthozoa: Hexacorallia: Scleractinia), having a solid calcareous skeleton, constitute the structural basis of a coral reef. Octocorals (Cnidaria: Anthozoa: Octocorallia) are ecologically diverse and important members of a wide range of marine communities, from shallow tropical to deep-sea coral reefs, where they provide additional habitat complexity and support biodiversity [1]. Gorgonians, a type of Octocorallia, are sessile colonial soft corals found throughout the world’s oceans, exhibit diverse morphologies and are an integral part of coral reefs [2,3]. This group of corals is characterized by a keratin-like axial skeleton consisting of the protein gorgonin [4].

Like all multicellular organisms, corals are holobiont organisms forming intricate and complex interactions with diverse microorganisms such as dinoflagellates, fungi and bacteria [5]. Intracellular microalgae (dinoflagellates of the family Symbiodiniaceae) are the best-known coral symbionts, that provide the coral host with nutrients through photosynthesis [6]. This symbiosis is crucial in adapting to changing environmental conditions [7]. The coral holobiont is sensitive to thermal stress, which causes coral bleaching (loss of symbionts). To date, coral reef bleaching events have become widespread [8]. To survive adverse environmental conditions, the flexibility of a coral host in associating with different Symbiodiniaceae endosymbionts can be critical. Corals usually contain one dominant strain of Symbiodiniaceae along with a background population of additional Symbiodiniaceae types [9]. Such diversity in the symbiont community harbored by corals can be beneficial during changing environmental conditions, allowing corals to acclimate by shuffling their symbionts towards dominance of a strain that is better adapted to the new conditions [10]. This flexibility in symbiosis, demonstrated by some corals, provides greater resilience to adverse conditions, although the symbiont community typically returns to its natural composition once the stress subsides [10].

In this study, we collected colonies of the photosynthetic gorgonian coral *Junceella fragilis* (Octocorallia, Scleralcyonacea, Ellisellidae) from the wild (coastal waters of Vietnam) and cultivated them in controlled conditions for a year. We studied the dinoflagellate assemblage of *J. fragilis* from the wild and after cultivation. The plasma membrane, which hosts numerous functionally diverse proteins, plays a critical role in maintaining cell shape and serves as the primary barrier against environmental stressors. Under normal environmental conditions, the membrane lipidome is in a stable state (homeostasis), which is manifested as a certain and constant ratio of membrane lipids and their structure [11]. The structure and mechanical properties of the plasma membrane are mainly determined by the molecular species profile of phospholipids (PLs) [12]. In corals, the major PLs are phosphatidylcholine (PC), phosphatidylethanolamine (PE), phosphatidylserine (PS), phosphatidylinositol (PI), and ceramideaminoethylphosphonate (CAEP) [13,14]. In dinoflagellates, the ether forms of PC, PE, PS, PI, and CAEP are absent in the membranes, which makes them the major markers of host coral tissues [15]. Photosynthetic membranes of Symbiodiniaceae are rich in glycolipids (GL) essential for their photosynthetic apparatus such as sulfoquinovosyldiacylglycerol (SQDG), monogalactosyldiacylglycerol (MGDG), and digalactosyldiacylglycerol (DGDG) [15,16]. The plasma membrane of dinoflagellates consists of diacyl forms of PC, PE, PI and betaine lipid (BL) 1,2-diacylglyceryl-3-O-carboxy (hydroxymethyl)-choline (DGCC) [17,18].

In this study, we aimed to determine whether the lipidomic profiles of the host and its symbionts, along with the symbiont community composition, would remain stable or shift in response to the altered environmental conditions of the aquarium. We hypothesized that the host plasma membrane would show higher plasticity compared to the more conserved thylakoid membranes of the symbionts. In this way, we carried out the comparative analysis of membrane lipids (PL, GL and BL) of the *J. fragilis* colonies from the wild and after cultivation by high-performance liquid chromatography with tandem mass spectrometry (HPLC-MS/MS).

## 2. Materials and Methods

### 2.1. Specimen Collection

Colonies of the tropical gorgonian coral *J. fragilis* (subclass Octocorallia, order Scleralcyonacea, family Ellisellidae) were collected by divers in the Gulf of Thailand, in the coastal waters of Vietnam, South China Sea (9°19.1′ N, 103°32.1′ E). These corals were found at a depth of 4–6 m and a temperature of 28 °C during a marine expedition aboard the R/V *Akademik Oparin* in August 2018. During the expedition we did not measure the level of the (photosynthetically active radiation) PAR; for this we used the following literary data [19,20]. We calculated the PAR for this region and depth: ~500 µmol/m^2^/s. We obtained lipid extracts from three gorgonian colonies using the method described below (see Section 2.4) in the onboard laboratory. In addition, we brought another three colonies to the aquarium facility of the NSCMB FEB RAS and cultivated for 12 months. We kept the colonies of gorgonian in a 500 L tank under the following conditions: aerated seawater connected to a flow-through circulation system (with seawater comes from Peter the Great Bay, Amur Bay, Russia, 43°19.8′ N, 131°91.8′ E), temperature—26 °C, salinity—35‰, pH—8.1 and nutrients (NO_3_^−^—5 mg/L, PO_4_^−3^—0.25 mg/L). We measured nutrient levels using the Salifert test systems (Salifert, Duiven, The Netherlands). We illuminated the colonies with a metal halide lamp fitted with a 250 W Aqualine 10,000 burner (16,000 Kelvin), PAR 200 µmol/m^2^/s (Aqua Medic, Bissendorf, Germany). The photoperiod was set at 12 h light/12 h dark. We then stored the colonies in individual 50 mL tubes with 70% ethanol until further genetic processing.

### 2.2. DNA Extraction, PCR and Sequencing

We extracted DNA from each coral colony according to the guanidine thiocyanate-based protocol published previously [21]. Six colonies (3 wild and 3 cultivated) were twice analyzed using the universal and genus-specific primers. In addition, we sequenced an approximately 920 bp fragment of the mitochondrial cytochrome *c* oxidase subunit I (CO1) gene using primers COII8068F (5′-CCATAACAGGACTAGCAGCATC-3′) [22] and COIOCTR (5′-TCATAGCATAGACCATACC-3′) [23]. We amplified nuclear large-subunit 28S ribosomal DNA (28S rDNA) using the primers 28S-Far (5′-CACGAGACCGATAGCGAACAAGTA-3′) and 28S-Rar (5′-TCATTTCGACCCTAAGACCTC-3′) [24]. We verified presence and specific types of Symbiodiniaceae by PCR screening and sequencing of the obtained products following the protocols and molecular markers published by Sikorskaya et al. [21,25]. The primer pairs used in the reported analysis and the PCR conditions are listed in Table 1.

We performed amplification reactions on a thermal cycler (Applied Biosystems Veriti 96 Well, Waltham, MA, USA) using two different DNA polymerases: the QIAGEN Taq PCR Master Mix (QIAGEN, Hilden, Germany) and the DreamTaq DNA polymerase (Thermo Fisher Scientific, Waltham, MA, USA) according to the manufacturer’s instructions.

Enzymatic PCR cleanup was treated with Exonuclease I (Exo I) and Shrimp Alkaline Phosphatase (rSAP) (New England Biolabs, Ipswich, MA, USA). Sequencing was performed using a Big Dye Terminator v3.1 Cycle Sequencing Kit (Perkin-Elmer, Foster City, CA, USA) according to manufacturer’s protocol. After the sequencing reaction, we removed the primers and unreacted dye using Centri-Sep™ columns with Sephadex G-50 (Princeton Separations, Inc. Adelphia, NJ, USA). We analyzed sequencing products on an ABI PRISM 3500 Genetic Analyzer (Applied Biosystems, Foster City, CA, USA).

### 2.3. Sequence Alignment and Molecular Phylogenetic Analysis

We proofread sequence data using the Geneious Prime software (v.2022.0.2, Biomatters Limited, Auckland, New Zealand). The sequences were compared with those accessed from the GenBank database (http://www.ncbi.nlm.nih.gov/, accessed on 20 January 2024) using the BLAST tool version 2.15.0 [34]. We deposited the obtained sequences in GenBank under the accession numbers: PQ799424-PQ799426, PQ805420-PQ805426, PQ805417-PQ805419, PQ836327, PQ836328, PQ858226, PQ858227, PQ859447-PQ859449. We aligned sequences using MAFFT v7 [35] on https://mafft.cbrc.jp/alignment/server/ (accessed on 20 January 2024). For this, we used default settings, with the alignment mode Q-INS-i employed and visually checked to exclude ambiguous regions which are difficult to align. The sequence of *Primnoa pacifica* (Primnoidae) was used as outgroup in the CO1 dataset. Phylogenetic trees were inferred from maximum likelihood (ML) and Bayesian inference (BI) analysis. The trees were estimated with nucleotide substitution models selected using ModelFinder [36] as implemented in the IQ-TREE program [37] under the corrected Akaike information criterion (AICc). Branch supports were assessed using the following options: bootstrap method based on 1000 ultra-fast bootstrap (UFBoot2) replicates [38] with a 0.99 minimum correlation as convergence criterion, 1000 replicates of the SH-aLRT (approximate likelihood ratio test [aLRT] and Shimodaira–Hasegawa [SH]-aLRT) branch test [-alrt] [39] and the Approximate Bayes test (-aBayes; Ref. [40]). The CO1 dataset included 16 sequences with 787 columns, 58 distinct patterns, 17 parsimony-informative, and 740 constant sites. The best-fit model of character evolution was TPM3u+F+I (0.779).

The dataset on the D1–D2 region of 28S rDNA consisted of 30 sequences of *Cladocopium*, *Breviolum* and *Gerakladium*, including the sequence of *Halluxium pauxillum* MT015652 as the outgroup. The Integer NJ Net algorithm of PopART v.1.7.2 software package [41] was used for the D1–D2 region of 28S rDNA network analysis. In this study, we selected the D1–D2 domain of the large 28S rDNA and the CO1 gene as targets for coral species identification. We investigated the molecular-genetic diversity of Symbiodiniaceae associated with three wild and three cultivated colonies of *J. fragilis* using the nuclear ITS1-5.8S-ITS2 rDNA typing, ITS2 type profiling, and 28S rDNA sequences.

### 2.4. Lipid Analysis

For lipid extraction, we used hexane, benzene, chloroform and methanol of analytical grade. For high-performance liquid chromatography with tandem mass spectrometry (HPLC-MS/MS), we used hexane, 2-propanol, formic acid (HCOOH), triethylamine (Et_3_N) of LC-MS grade (Sigma-Aldrich, Saint Louis, MO, USA).

We obtained total lipid extracts according to [42] with some modifications. A sample (~1 g) of fresh coral colonies was homogenized in 3 mL of a chloroform:methanol (2:1 *v*/*v*) mixture and filtered. Residues were extracted twice with 3 mL of a chloroform:methanol (2:1 *v*/*v*) mixture. The extracts were combined, mixed with chloroform (3 mL) and water (3.5 mL), and then left overnight for phase separation at 4 °C. Then the near-bottom layer was separated and evaporated on a rotary evaporator; the total lipids were dissolved in a small volume of chloroform under argon and stored at −40 °C.

We analyzed the contents and structures of PL, GL and BL molecular species on a HPLC system with a high-resolution tandem mass spectrometer (Shimadzu, Kyoto, Japan) [43]. Total lipids were separated on a Shim-Pack diol column (4.6 mm × 50 mm, particle size 5 μm) (Shimadzu, Kyoto, Japan) using a Nexera-e chromatography system (Shimadzu, Kyoto, Japan). Solvent system A (2-propanol:hexane:H_2_O:HCOOH:(28%)NH_4_OH:Et_3_N, 28:72:1.5:0.1:0.05:0.02, *v*/*v*) and solvent system B (2-propanol:H_2_O:HCOOH:(28%)NH_4_OH:Et_3_N, 100:1.5:0.1:0.05:0.02, *v*/*v*) were used as eluents. The percentage of system B in the total solvent flow was programmed as follows: 0 to 20% (7 min), 20 to 100% (5 min), 100% (5 min), 100 to 0% (0.1 min), and 0% (10 min). The flow rate was 0.2 mL/min. Lipids were detected on a high-resolution tandem mass spectrometer LCMS-IT-TOF (Shimadzu, Kyoto, Japan). Analysis was performed in the electrospray ionization (ESI) mode with simultaneous registration of signals of positive and negative ions. Scanning was performed in a *m*/*z* range of 100–1200. The source voltage was −3.5 kV in the case of negative ion formation and 4.5 kV in the case of the formation of positive ions. The temperature of the ion source was 250 °C; dry gas (N_2_) pressure, 200 kPa; the flow rate of nebulizing gas (N_2_), 1.5 L/min. Argon (0.003 Pa) was used in the collision chamber of the mass spectrometer. Percentages of certain molecular species of each lipid class were calculated on the basis of the peak area of negative ions [M–H]^−^, except PC, MGDG, DGDG and DGCC which were determined using the peak area of positive ions [M+H]^+^ and [M+Na]^+^. Molecular species were identified as described earlier [13]. The identification of selected molecular species is presented in the Supplementary Materials (Figures S1–S4).

Lipid molecular species were designated using an abbreviated nomenclature based on the LIPID MAPS recommendations [44]. Each lipid species was identified by its class (Phospholipids: PC, PE, PS, PI; Phosphonolipid: CAEP; Glycolipids: MGDG, DGDG, SQDG; Betaine lipids: DGCC) followed by the composition of its two hydrophobic chains. For phospholipids, the chain-1/chain-2 format is used, where the first chain (*sn-1* position) is defined by the ratio of the number of carbon atoms to the number of double bonds, with a suffix indicating the bond type (no suffix for ester bond, “e” for ether bond), and the second chain (*sn-2* position) is defined by the ratio of carbon atoms to double bonds (always ester bonds in the lipids studied). For phosphonolipids, the “sphingoid base/fatty acid” format is used, where “b” denotes the sphingoid base. Glycolipids and betaine lipids also use the “chain-1/chain-2” format, as with phospholipids.

### 2.5. Statistical Analysis

Values of lipid contents are presented as mean ± standard deviation for three biological samples. Raw data were used after being tested for normality of distribution (Shapiro–Wilk’s test). Significant differences between levels within the factors were determined by the analysis of variance (ANOVA) with the post hoc Tukey’s HSD test. A probability level of *p* < 0.05 was considered statistically significant. The independent variables were wild and cultivated groups of *J. fragilis*; response variables were contents of PL, GL and BL molecular species. The raw data was arcsine-transformed [45] prior to building of the heat maps and to the principal component analysis (PCA) (Tree Clustering, Ward’s Method and Euclidean Distances). The heat maps were created, and all statistical analyses performed using the R statistical software version 4.4.1 (“rstatix” package for ANOVA and the post hoc Tukey’s HSD test, and “pheatmap” package for heat maps).

## 3. Results

### 3.1. Molecular Phylogenetic Analyses

**Figure 1:**
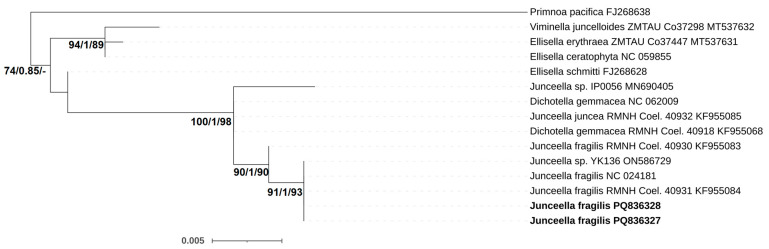
Phylogenetic position of wild and cultivated *Junceella fragilis* and relationships among the closest Ellisellidae species, as inferred from SH-aLRT test, Bayesian, and Maximum likelihood analyses of CO1 mtDNA sequences. The SH-aLRT support (%), the aBayes support, and ultrafast bootstrap support (%) values over 70 and over 0.7 are shown at the nodes. The scale bar represents inferred evolutionary distance in changes/site. Bold letters indicate sequences generated in this study.

As shown by analysis of the CO1 mtDNA sequences, the *Junceella* specimens shared 100–99.8% sequence similarity to the *J. fragilis* sequences (KF955084, NC_024181, ON586729 and KF955083) from NCBI. The 28S rDNA sequence analysis showed that all the *J. fragilis* samples had 99.6 to 100% similarity (1 bp difference) to *J. fragilis* (AF263355 and MZ190000), which confirmed the morphological description. The sequences of *J. fragilis* from this study and from GenBank are grouped together into a single clade with high support (Figure 1).

**Figure 2:**
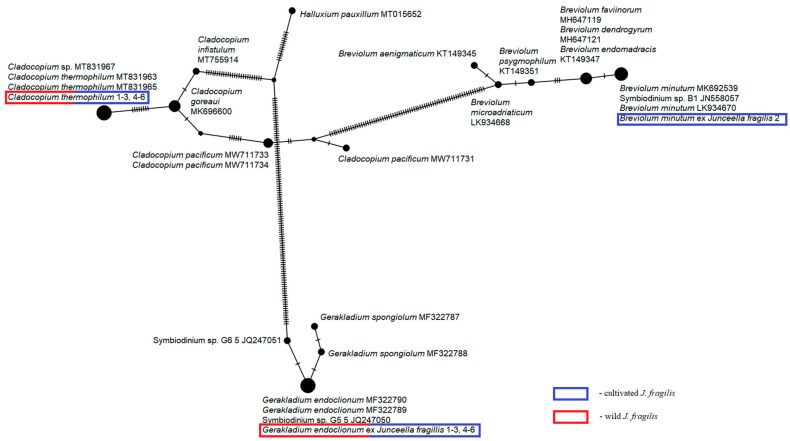
Integer NJ Net network is based on the D1–D2 region of 28S rDNA from 30 sequences of *Cladocopium*, *Breviolum* and *Gerakladium*. Mutational steps are symbolized by dashes; the diameter of the circles is proportional to the number of sequences that belong to each ribotype. Sequences obtained in this study are outlined by red and blue rectangles.

Our study revealed the presence of two species (*Cladocopium thermophilum* and *Gerakladium endoclionum*) in all wild and cultivated *J. fragilis* colonies. The sequences of *Cladocopium* were aligned with those of *Cladocopium* C3 and *C. thermophilum* from the NCBI database using Geneious R11. All sequences of the ITS2 region-specific to the *C. thermophilum* clade were checked for the presence or absence of the 8-base pair “S. thermo.-indel” [46]. The specific indel was detected in the analyzed ITS2 sequences. In addition, *Breviolum minutum* was detected in only one of the cultivated *J. fragilis* colony. The Integer NJ Net network based on the 28S rDNA dataset from *Cladocopium, Breviolum* and *Gerakladium* sequences obtained this study and accessed from GenBank is presented in Figure 2.

As shown in the analyses of [47,48,49], dinoflagellates of clade G (*Gerakladium* spp.) have been previously identified in *J. fragilis* individuals. We have previously identified *Cladocopium* spp. in *J. fragilis* colonies [13].

### 3.2. Polar Lipidome of the Gorgonian J. fragilis

Using HPLC-MS/MS, we identified 47 molecular species of PL, of which 15 were PE molecular species, 11—PC molecular species, 7—PS molecular species, 8—PI molecular species, and 6—CAEP molecular species (Table S1). The predominant molecular species of PL of *J. fragilis* (defined as those accounting for >10% of their respective PL class) were PC 16:0e/20:4, PC 18:0e/20:4; PE 18:1e/20:4, PE 19:1/20:4, PE 18:1e/24:6; PS 18:1e/22:4, PS 18:0e/22:4, PS 18:0/22:4; PI 18:0/22:4; CAEP 18:1b/16:0, CAEP 18:1b/16:1.

**Figure 3:**
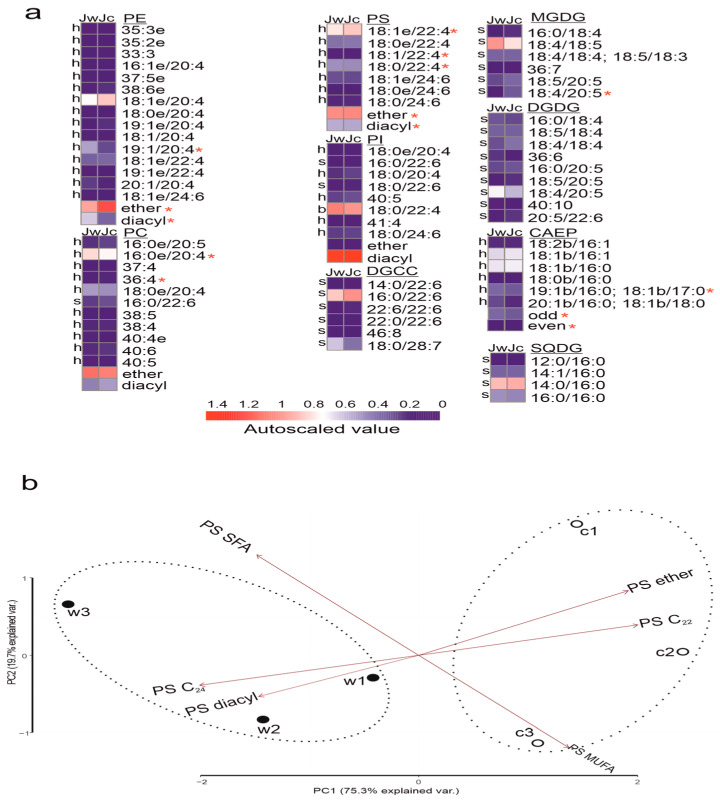
(**a**). Heat map of lipid molecular species in the symbiotic gorgonian soft coral *Junceella fragilis*. The scale bar under the heat map represents the arcsine-transformed relative abundance of lipid content in the samples. Content values (mean ± SD, n = 3 of biologically independent samples) are presented as percentage of the lipid class. Red asterisks indicate statistically significant differences * *p* < 0.05 (HSD test) between the two groups of wild *J. fragilis* (Jw) and *J. fragilis* (Jc) kept in artificial conditions in the lab. The notations “h”, “s” and “b” denote the source of a given molecular species: “h” is the host organism, “s” is the symbiont and “b” is the host organism and the symbiont. “Even” and “odd” denote the parity of the fatty acid or sphingoid base chain; “ether” indicates the presence of an ether linkage at the *sn-1* position; ‘diacyl’ denotes ester linkages at the *sn-1* and *sn-2* position. Glycerophospholipids that have an alkyl/alkenyl chain attached to the *sn-1* position by an ether bond are indicated as “e”. (**b**). A principal component analysis (PCA) of PS molecular species composition with different fatty acids (FAs): monounsaturated FA (MUFA), saturated FA (SFA), C22 PUFA, and C24 PUFA. The acronyms are as follows: phosphatidylcholine (PC), phosphatidylethanolamine (PE), phosphatidylserine (PS), phosphatidylinositol (PI), ceramideaminoethylphosphonate (CAEP), sulfoquinovosyldiacylglycerol (SQDG), mono- and digalactosyldiacylglycerol (MGDG and DGDG), 1,2-diacylglyceryl-3-O-carboxy-(hydroxymethyl)-choline (DGCC).

In *J. fragilis*, most of PC and PE molecular species had an ether bond in their structure (Figure 3a). In *J. fragilis,* lipids had a high abundance of PC and PE molecular species with C20 and C22 polyunsaturated fatty acids (PUFA). It should be noted that the molecular species PE 18:1e/24:6 included the tetracosapolyenoic fatty acid (TPA) 24:6(n-3). Both wild and cultivated *J. fragilis* colonies displayed significant variations in contents of PC 16:0e/20:4, PC 36:6; PE 19:1e/20:4, ether PE and diacyl PE molecular species (HSD test, *p* < 0.05). In addition, only cultivated corals had a molecular species PE 19:1e/22:4.

*Junceells fragilis* showed higher contents of PS and PI molecular species with C22 and C24 PUFA. The PS molecular species were almost entirely in the ether form, unlike the PI molecular species that were mainly in the diacyl form. The wild and cultivated *J. fragilis* differed significantly in the contents of PS 18:1e/22:4, PS 18:1/22:4, PS 18:0/22:4, ether PS and diacyl PS molecular species. We detected molecular species of PI that were characteristic of the symbionts, the host, and both. PI molecular species with 22:6 PUFA were found only in symbionts; those with 22:4 PUFA—in both, and those with 20:4 PUFA—only in the host [50]. Wild and cultivated *J. fragilis* did not significantly differ in the contents of PI molecular species. Figure 3b shows the PCA of corals based on the composition of PS molecular species with different FA: PS with monounsaturated FAs (MUFA), saturated FAs (SFA), C22 PUFAs, and C24 PUFAs. The wild and cultivated coral colonies clearly split into two clusters (Tree Clustering, Ward’s Method and Euclidean Distances). The wild *J. fragilis* colonies were shifted along the PC1 axis, primarily due to the higher content of PS molecular species with C24 PUFAs and PS diacyl, while in the cultivated group was dominated by PS ether and PS with C22 PUFA.

We identified a total of six molecular species of CAEP in the studied gorgonians. The molecular species with 16:0 (palmitic acid) linked to monoenoic and diene sphingoid bases were the major in *J. fragilis*. In addition to sphingoid bases 18:1b and 18:2b, ceramide components containing other dihydroxylated bases such as 18:0b, 19:1b, and 20:1b were also detected. The content of 19:1b/16:0, 18:1b/17:0 and odd-number CAEP molecular species was significantly higher in wild than in cultivated *J. fragilis* (Figure 3a).

### 3.3. Lipidome of Symbionts

In the total lipid extract, we identified 19 GL molecular species and 6 BL molecular species BL, all of which belonged to the DGCC class. The major molecular species of GL of *J. fragilis* (defined as those accounting for > 10% of their respective GL class) were as follows: MGDG 18:4/18:5, MGDG 18:4/18:4, MGDG 18:5/18:3; DGDG 18:5/18:4; DGDG 18:4/20:5, DGDG 16:0/20:5; SQDG 14:0/16:0, SQDG 16:0/16:0, SQDG 14:1/16:0; DGCC 16:0/22:6, and DGCC 18:0/28:7 (Table S2).

Most MGDG detected in the fragment contained a fatty acyl tail of 18:4. In the cultivated gorgonians, the content of the molecular species MGDG 18:4/20:5 was significantly increased compared to wild specimens (HSD test, *p* < 0.05). As in MGDG, the major molecular species in DGDG contained PUFA 18:4. There were no significant differences between the DGDG molecular species in the wild and cultivated gorgonians. SQDG primarily contained molecular species with FA 16:0 as one of the tails. No significant differences were observed in molecular species of SQDG between the wild and cultivated *J. fragilis*. Molecular species of DGCC contained docosahexaenoic acid (22:6), and there was one species containing a very-long-chain PUFA (28:7). However, no significant differences in the molecular species of DGCC were found between the wild and cultivated colonies.

## 4. Discussion

This study compared the lipidomes and symbiont communities of wild *J. fragilis* from the South China Sea with colonies maintained for one year in a controlled aquarium system. Understanding the effects of such a habitat shift is crucial, as many laboratory experiments on coral physiology rely on aquarium-acclimatized specimens. Our results reveal a remarkable stability in the symbiont community and their thylakoid lipids, coupled with significant remodeling of the host’s plasma membrane phospholipids, particularly ether-linked species.

*Cladocopium thermophilum* was first discovered and described in the southern Persian Gulf [46]. Oladi et al. [51] reported further detections of the species in the northern Persian Gulf and the Gulf of Oman. Recent studies in Indonesia have confirmed the presence of the heat-tolerant species *Cladocopium thermophilum* (previously classified as type C3) in the waters off the coasts of Java and Bali. Genetic markers (ITS2 and psbA) have shown 96.79–100% similarity to isolates from the Persian Gulf, particularly within the corals of the genera *Acropora* and *Porites* [52,53]. In this study, colonies of coral *J. fragilis*, in which *Cladocopium thermophilum* was discovered, were collected in the Gulf of Thailand, in the coastal waters of Vietnam, South China Sea. This is the first time this species has been found in this area. These findings suggest that *C. thermophilum* is a pan-Indo-Pacific thermophilic specialist rather than a narrow endemic that thrives in high-temperature environments.

Ether-linked PC species with PUFA 20:4 (host origin) [50] dominated the PL profiles of *J. fragilis*, while symbiont-specific PC 16:0/22:6 was also detected. Most PC molecular species did not differ significantly between wild and cultivated colonies, with the exception pf PC 16:0e/20:4 and PC 36:4, which showed significant differences (Figure 3a). However, cultivated colonies had higher content of ether PE but a lower content of diacyl PE (particularly 19:1/20:4) than wild colonies. The molecular species with odd number FA (PE 19:1/20:4, 19:1e/20:4, 19:1e/22:4) indicates the presence of bacteria in *J. fragilis* [54]. This suggests a greater contribution of the bacterial community in the wild gorgonian colonies compared to those kept in artificial conditions. The predominance of n-6 PUFAs, particularly 20:4 and 22:4, in PC and PE is a characteristic feature of shallow-water gorgonians including *J. fragilis* [55]. PS and PI are anionic PL, which are essential structural components of membranes, act as signaling molecules, interact with proteins, and can influence membrane dynamics and remodeling [56]. Ether-linked PS represents a rare subclass of PL whose functions remain poorly known, with limited data available. Gorgonians contain numerous ether-linked PS molecular species, and a significant increase in the predominant PS species was observed in the cultivated *J. fragilis*. This difference may reflect adaptation to light intensities in aquarium conditions, which differed markedly from the illumination at great depths where wild gorgonians typically reside. We observed a significant increase in the levels of ether-linked PS and PE in cultivated *J. fragilis* compared to wild colonies (Figure 3a). This increase may reflect membrane adaptation to altered light conditions, as ether PL are known to confer antioxidant properties and enhance membrane resistance to lipolytic enzymes [57]. Light intensity is known to influence the composition of PL, namely, PC, PE, and PS, in photosynthetic organisms, including membrane lipid remodeling in response to oxidative stress [58]. PS included molecular species containing PUFA 24:6, a known chemotaxonomic marker of the subclass Octocorallia [59]. We did not observe significant variations in the PI molecular species profile between the wild and cultivated colonies. This may indicate that the *J. fragilis* colonies were not subject to bleaching either in the wild or in the tank. A sharp decrease or complete absence of molecular species of PI is an indicator of the coral bleaching process [60,61]. In addition to light intensity, the temperature difference between wild (28 °C) and cultivated (26 °C) conditions may have also influenced *J. fragilis* PL composition, as membrane fluidity and lipid saturation are known to respond to temperature variation [11].

CAEP is a sphingolipid with 2-aminoethylphosphonate as a polar head group [62]. Like PL, CAEP is one of the major structural components of the cell membrane of marine invertebrates [14,15,63]. In some cases, its content is comparable to the major lipid classes, PC and PE [15]. We observed a significant reduction in the contents of CAEP molecular species 19:1b/16:0 and 18:1b/17:0 in cultivated gorgonians compared to the wild specimens. However, data on the functions of CAEP in marine invertebrates are limited, which highlights the need for further research.

Thylakoid membranes of dinoflagellate photosynthetic systems are mainly composed of GL, including MGDG, DGDG, and SQDG [16]. Two neutral (uncharged at physiological pH) major classes, MGDG and DGDG, contain one or two galactose residues in their polar head, respectively [64]. These lipids have contrasting physicochemical properties: DGDG forms bilayers, whereas MGDG does not [65]. There were no significant differences in the molecular species of DGDG and SQDG, but there was noticeable difference in the content of one molecular species—MGDG (18:4/20:5). BL are a class of acylglycerolipids that have a quaternary amine alcohol ether-linked to a diacylglycerol moiety and lack in phosphorus [66,67]. These lipids typically have a zwitterion structure resembling that of PC, suggesting that these two components perform the same or similar structural function (form lamellar phase) in the membrane [68]. We found no significant differences in the GL and BL molecular profiles between the wild and cultivated *J. fragilis* colonies. As previously noted, both wild and cultivated colonies maintained nearly identical compositions of symbiotic dinoflagellates.

## 5. Conclusions

Notably, the corals translocated from the South China Sea and maintained under artificial conditions for one year have not shown any significant shifts in their symbiotic algal composition. Previously, there were no reports of several species of Symbiodiniaceae found simultaneously in corals of the genus *Junceella*. In our study, we recorded the presence of a few Symbiodiniaceae species in all *J. fragilis* colonies. However, only one of the colonies was found *B. minutum*. This symbiotic relationship is fundamental to the productivity of tropical cnidarians, as dinoflagellates translocate photosynthetically fixed carbon to the host in the form of organic compounds, supporting the host’s energy demands and influencing its overall lipid metabolism. The fact that the symbiotic community has not changed dramatically is also confirmed by the absence of significant differences between the lipid profile of the thylakoid membranes of symbionts of wild colonies of *J. fragilis* and those after exposure to artificial conditions. As was shown [25], differences in thylakoid lipidome are already observed at the species level, which is consistent with the results of this work. At the same time, we observe differences in the profile of the molecular species of plasma membrane lipids between wild and cultivated corals. The structural features of the molecular species of coral PL are their chemotaxonomic characteristics, and the molecular profile of these lipids depends on various factors [15]. The plasma membrane is the first barrier to the adverse effects of environmental factors, and the profile of molecular species, primarily PL, of the membrane is very sensitive to light intensity and temperature, which is probably the reason for the differences found in the profiles of molecular PL species in this work.

## Figures and Tables

**Table 1:** Primers and amplification conditions used in this study to screen and identify Symbiodiniaceae in wild and cultivated colonies of *Junceella fragilis*.

Gene	Primer Name	Primer Sequence (5′ → 3′)	Program	Reference
Zooxanthellae				
28S rRNA (D1-D2)	28Szoox-D1/D2F 28Szoox-D1/D2R	CCTCAGTAATGGCGAATGAACA CCTTGGTCCGTGTTTCAAGA	96 °C—3 min (96 °C 60 s, 55 °C 60 s, 72 °C 120 s) ×5, (96 °C 30 s, [55 → 50] °C 60 s, 72 °C 120 s) ×30, 72 °C 7 min	[26]
ITS2	ITSintfor2 ITS2clamp or ITS-Reverse	GAATTGCAGAACTCCGTG CGCCCGCCGCGCCCCGCGCCCGTCCCGCGG GATCCATATGCTTAAGTTCAGCGGGT GGGATCCATATGCTTAAGTTCAGC GGGT	96 °C—3 min (96 °C 30 s, [62 → 52] °C 30 s, 72 °C 120 s) ×20, (92 °C 30 s, 52 °C 30 s, 72 °C 120 s) ×20, 72 °C 10 min	[27] [28]
ITS region	zITSf zITSr	CCGGTGAATTATTCGGACTGACGCAGTGCT TCCTCCGCTTATTGATATGC		[29] [30]
ITS region clade A-specific	A (forward) A (reverse)	CCTCTTGGACCTTCCACAAC GCATGCAGCAACACTGCTC	96 °C—5 min (96 °C 30 s, 60 °C 30 s, 72 °C 60 s) ×38, 72 °C 5 min	[31]
28S rRNA (D2) clade B-specific	B (forward) B (reverse)	GTCTTTGTGAGCCTTGAGC GCACACTAACAAGTGTACCATG		
28S rRNA (D2) clade C-specific	C (forward) C (reverse)	CTTGAAATCGCTGAAAGGGA CTATTCACGCTTAAGCACACA		
28S rRNA (D2) clade D-specific	D (forward) D (reverse)	GCCGTGTACGGTGCTCGCTCTCAA GGCCACTCGCAAATGGACAGC		
ITS2 A-specific	S.S. ITS2 F S.S. ITS2 R	TTCTGCTGCTCTTGTTATCAGG ACACACATGAGCTTTTGTTTCG	96 °C—3 min (96 °C 30 s, [62 → 52] °C 30 s, 72 °C 120 s) ×20, (92 °C 30 s, 52 °C 30 s, 72 °C 120 s) ×20, 72 °C 10 min	[32]
ITS2 B-specific	S.B. ITS2 F S.B. ITS2 R	GCAAGCAGCATGTATGTC CTTGGAACAACAGTACGCTC		
ITS2 C-specific	S.C. ITS2 F S.C. ITS2 R	TGCGTTCTTATGAGCTATTGCC CAGCGTCACTCAAGTAAAACCA		
ITS2 D-specific	S.D. ITS2 F S.D. ITS2 R	TTTGCTTCAGTGCTTATTTTACCT ACGGCGCAGAAGGACAC		
ITS2 E-specific	S.E. ITS2 F S.E. ITS2 R	GAGGTAAGCTGGACTGATTTG TTAGTTCCTTTTCCTCCGCT		
ITS2 F-specific	S.F. ITS2 F S.F. ITS2 R	CCTGTGAGCCATTGAAACTCTAGT CAGCGTCACTCAAGAAATACCAT		
ITS2 G-specific	S.G. ITS2 F S.G. ITS2 R	CAGTGCAATGCCTCCTTGTG CCCACGCATATTCCGGAGA		
28S rRNA (D2) clade A-specific	SymA-28S F SymA-28S R	GATTGTGGCCTTTAGACATACTACC CTCTGAGAGCAAGTACCGTGC	96 °C—3 min (96 °C 30 s, [62 → 55] °C 30 s, 72 °C 120 s) ×20, (92 °C 30 s, 55 °C 30 s, 72 °C 120 s) ×20, 72 °C 10 min	[33]
28S rRNA (D2) clade B-specific	SymB-28S F SymB-28S R	CACATGTCGTGCTGAGATTGC CTCGCATGCTGAGAAACACTG		
28S rRNA (D2) clade C-specific	SymC-28S F SymC-28S R	TTGCTGAGATTGCTGTAGGCT TCCTCAAACAGGTGTGGC		
28S rRNA (D2) clade D-specific	SymD-28S F SymD-28S R	AATGCTTGTGAGCCCTGGTC AAGGCAATCCTCATGCGTATG		
28S rRNA (D2) clade E-specific	SymE-28S F SymE-28S R	CGAGTTTTCACTAGCCTTGTGTG AGCGTTGCAGCTGACGAG		
28S rRNA (D2) clade F-specific	SymF-28S F SymF-28S R	ACAGATCTTGCTGAGATTGCTGTG GAAGGCCGTCCTCAAACAGAC		

## Data Availability

The original contributions presented in this study are included in the article/Supplementary Materials. Further inquiries can be directed to the corresponding author.

## Supplementary Materials

The following supporting information can be downloaded at https://www.mdpi.com/article/10.3390/biology15080602/s1, Table S1: Molecular species of phospholipids (PL) wild and cultivated *Junceella fragilis*; Table S2: Molecular species of glycolipids (GL) and betaine lipids (BL) in wild and cultivated *Junceella fragilis*; Figure S1 Electrospray ionization mass spectra of proposed 19:1e/20:4 PE; Figure S2 Electrospray ionization mass spectra of proposed 18:0e/20:4 PC; Figure S3 Electrospray ionization mass spectra of proposed 18:1e/22:4 PS; Figure S4 Electrospray ionization mass spectra of proposed 18:0/22:4 PI.

## Abbreviations

The following abbreviations are used in this manuscript:

PL	Phospholipid
PC	Phosphatidylcholine
PE	Phosphatidylethanolamine
PS	Phosphatidylserine
PI	Phosphatidylinositol
CAEP	Ceramideaminoethylphosphonate
GL	Glycolipids
SQDG	Sulfoquinovosyldiacylglycerol
MGDG	Monogalactosyldiacylglycerol
DGDG	Digalactosyldiacylglycerol
BL	Betaine lipid
DGCC	1,2-diacylglyceryl-3-O-carboxy (hydroxymethyl)-choline
HPLC-MS/MS	High-performance liquid chromatography with tandem mass spectrometry
Exo I	Exonuclease I
rSAP	Shrimp Alkaline Phosphatase
ML	Maximum likelihood
AICc	Akaike information criterion
UFBoot2	1000 ultra-fast bootstrap
CO1	Mitochondrial cytochrome c oxidase subunit I
ESI	Electrospray ionization
ANOVA	Analysis of variance
PCA	Principal component analysis
FAs	Fatty acids
PUFA	Polyunsaturated fatty acids
TPA	Tetracosapolyenoic fatty acid
MUFA	Monounsaturated FAs
SFA	Saturated FAs
