# Supplementary material for: Influence of Habitat Alteration on the Molecular Profile of Membrane Lipids of the Coral Junceella fragilis
Source: Biology (Basel). 2026 Apr 10;15(8):602. doi: 10.3390/biology15080602 (PMC13113471; doi:10.3390/biology15080602)
Supplement: Supplementary file 1 [file biology-15-00602-s001.zip › biology-4165426-supplementary.pdf]

Table S1. The content of molecular species of phospholipids of *Junceella fragilis*

Molecular species of PL				
		Wild J. fragilis	Cultivated J. fragilis	p value
Phosphatidylinositols (% from total PI)				
1	18:0e/20:4 PI	1.272±0.728	2.041±1.327	0,428535879
2	16:0/22:6 PI	1.396±1.08	2.144±0.96	0,420915967
3	18:0/20:4 PI	4.172±0.496	6.098±1.176	0,0593476365
4	18:0/22:6 PI	2.12±0.761	3.39±0.818	0,12039576
5	40:5 PI	6.7±1.483	7.695±2.011	0,528284383
6	18:0/22:4 PI	76.733±4.085	70.297±3.52	0,107722756
7	41:4 PI	1.656±0.442	1.926±0.517	0,529273731
8	18:0/24:6 PI	5.952±1.34	6.408±2.282	0,780391457
9	Ether-PI	1.272±0.728	2.041±1.327	0,428535879
10	Diacyl-PI	98.728±0.728	97.959±1.327	0,428535879
11	Odd PI	1.656±0.442	1.926±0.517	0,529273731
12	22:4-PI	76.733±4.085	70.297±3.52	0,107722756
13	22:6-PI	3.516±1.84	5.534±1.758	0,241675136
Phosphatidylserines (% from total PS)				
1	18:1e/22:4 PS	49.786±3.934	57.056±4.848	0,0489788957
2	18:0e/22:4 PS	14.067±3.311	15.57±4.274	0,866206601
3	18:1/22:4 PS	2.327±0.591	1.804±1.324	0,00455129378
4	18:0/22:4 PS	21.353±1.331	19.864±3.648	0,0213608217
5	18:1e/24:6 PS	8.063±0.571	7.126±1.706	0,0921003094
6	18:0e/24:6 PS	0.877±0.187	1.186±0.586	0,293910646
7	18:0/24:6 PS	3.527±0.659	3.76±0.27	0,44219066
8	Ether-PS	72.793±1.995	74.572±5.029	0,00826321938
9	Diacyl-PS	27.207±1.995	25.428±5.029	0,00826321938
10	C_{22} PS	87.533±0.886	87.928±1.446	0,073119118
11	C_{24} PS	12.467±0.886	12.072±1.446	0,073119118
12	SFA-PS	39.824±2.852	40.38±2.495	0,22570109
13	MUFA-PS	60.176±2.852	59.62±2.495	0,22570109
Phosphatidylethanolamines (% from total PE)				
1	35:3e PE	0.166±0.149	0.348±0.364	0,467991954
2	35:2e PE	1.579±0.616	1.43±0.603	0,779234403
3	33:3 PE	0.859±1.488	0.021±0.036	0,384696631
4	16:1e/20:4 PE	1.393±1.293	2.665±1.881	0,389347596
5	37:5e PE	0.739±0.251	0.815±0.295	0,751366088
6	38:6e PE	1.735±0.331	4.476±2.897	0,17902686
7	18:1e/20:4 PE	45.115±3.202	55.339±13.708	0,277066223
8	18:0e/20:4 PE	2.038±1.173	2.837±0.538	0,344085239
9	19:1e/20:4 PE	2.599±0.497	5.357±2.11	0,0925383178
10	18:1/20:4 PE	2.158±0.918	0.437±0.597	0,0531250763
11	19:1/20:4 PE	24.469±4.611	7.404±8.479	0,0377365097
12	18:1e/22:4PE	10.57±2.374	11.906±3.35	0,603477327
13	19:1e/22:4 PE	0±0	2.936±1.835	0,0504542646
14	20:1/20:4 PE	5.93±1.372	3.212±2.317	0,155464276
15	18:1alk/24:6 PE	0.649±0.153	0.818±0.114	0,200042822
16	Ether-PE	66.583±6.637	88.926±4.027	0,00774140153
17	Diacyl-PE	33.417±6.637	11.074±4.027	0,00774140153
18	Odd PE	30.412±2.86	17.645±11.449	0,134420854
19	20:4-PE	81.103±4.786	72.56±3.546	0,0681016844
Phosphatidylcholines (% from total PC)				
1	16:0e/20:5 PC	4.276±1.036	6.358±1.065	0,120027806
2	16:0e/20:4 PC	52.035±1.971	46.829±2.396	0,0104256405
3	37:4 PC	2.862±0.882	1.858±0.772	0,154781583
4	36:4 PC	0.744±0.766	3.298±0.92	0,0245617404
5	18:0e/20:4 PC	23.627±5.575	20.562±2.502	0,521756964
6	16:0/22:6 PC	5.517±1.752	7.502±1.617	0,169899

7	38:5 PC	1.106±0.562	1.461±0.151	0,497277098
8	38:4 PC	3.266±1.706	3.737±0.546	0,745809492
9	40:4e PC	2.172±0.609	2.889±1.174	0,486017285
10	40:6 PC	4.021±1.394	4.974±0.963	0,437290804
11	40:5 PC	0.375±0.162	0.531±0.128	0,322070229
12	Ether-PC	82.11±3.841	76.639±2.12	0,212831634
13	Diacyl-PC	17.89±3.841	23.361±2.12	0,212831634
14	Odd PC	2.862±0.882	1.858±0.772	0,154781583
Ceramideaminoethylphosphonates (% from total CAEP)				
1	18:2b/16:1 CAEP	3.55±1.034	3.688±0.782	0,862250933
2	18:1b/16:1 CAEP	36.816±1.707	39.986±3.951	0,271384406
3	18:1b/16:0 CAEP	39.687±0.964	41.403±1.467	0,166072817
4	18:0b/16:0 CAEP	0.921±0.502	1.129±1.12	0,784205953
5	19:1b/16:0; 18:1b/17:0 CAEP	12.146±0.991	9.807±0.166	0,0158920264
6	20:1b/16:0; 18:1b/18:0 CAEP	6.879±1.867	3.987±1.741	0,121464858
7	18:1b/16:1 + 18:1b/16:0 CAEP	76.504±2.552	81.389±3.744	0,135462799
8	Odd CAEP	12.146±0.991	9.807±0.166	0,0158920264
9	Even CAEP	87.854±0.991	90.193±0.166	0,0158920264
10	C_{36} CAEP	6.879±1.867	3.987±1.741	0,121464858
12	SFA CAEP	0.921±0.502	1.129±1.12	0,784205953

Data are presented as mean ± SD, *n* = 3

Table S2. The content of molecular species of glycolipids and betaine lipids of *Junceella fragilis*

Molecular species of GL and BL				
		Wild J. fragilis	Cultivated J. fragilis	p value
Monogalactosyldiacylglycerols (% from total MGDG)				
1	16:0/18:4 MGDG	2.042±1.493	6.063±3.305	0,127361299
2	18:4/18:5 MGDG	71.128±3.971	52.565±14.723	0,102857331
3	18:4/18:4; 18:5/18:3 MGDG	13.293±4.069	13.115±2.917	0,95395764
4	36:7 MGDG	2.112±0.427	3.038±1.036	0,225730669
5	18:5/20:5 MGDG	8.828±1.648	13.876±0.859	0,00942864181
6	18:4/20:5 MGDG	2.598±0.379	11.343±6.983	0,0964631047
7	C_{16} MGDG	2.042±1.493	6.063±3.305	0,127361299
8	C_{18-22} MGDG	97.958±1.493	93.937±3.305	0,127361299
Digalactosyldiacylglycerols (% from total DGDG)				
1	16:0/18:4 DGDG	10,898±4,697	8,867±7,809	0,719278706
2	18:5/18:4 DGDG	12,249±20,634	12,78±21,226	0,976834289
3	18:4/18:4 DGDG	9,383±3,326	16,72±14,318	0,43631031
4	36:6 DGDG	6,719±1,066	3,572±2,3	0,0981278656
5	16:0/20:5 DGDG	8,559±8,138	10,171±5,316	0,788394942
6	18:5/20:5 DGDG	3,725±3,463	5,756±4,058	0,545990495
7	18:4/20:5 DGDG	43,902±19,778	31,392±24,972	0,533934771
8	40:10 DGDG	0,903±0,913	4,271±2,853	0,123448796
9	20:5/22:6 DGDG	3,663±3,739	6,47±7,846	0,605905113
10	C_{16} DGDG	19,457±5,105	19,038±12,321	0,959351866
11	C_{18-22} DGDG	80,543±5,105	80,962±12,321	0,959351866
Sulfoquinovosyldiacylglycerols (% from total SQDG)				
1	12:0/16:0 SQDG	2,842±1,615	2,045±0,477	0,458408786
2	14:1/16:0 SQDG	13,588±1,813	13,389±1,286	0,884317574
3	14:0/16:0 SQDG	61,088±3,606	64,439±4,381	0,364311947
4	16:0/16:0 SQDG	22,482±1,353	20,127±3,981	0,387165065
5	MUFA-SQDG	13,588±1,813	13,389±1,286	0,884317574
6	SFA-SQDG	86,412±1,813	86,611±1,286	0,884317574
7	C_{28-30} SQDG	77,518±1,353	79,873±3,981	0,387165065
8	C_{32} SQDG	22,482±1,353	20,127±3,981	0,387165065
9	C_{28} SQDG	2,842±1,615	2,045±0,477	0,458408786
10	C_{30-32} SQDG	97,158±1,615	97,955±0,477	0,458408786
Diacylglyceryl-3-O-carboxy-(hydroxymethyl)-cholines (% from total DGCC)				
1	14:0/22:6 DGCC	59.227±13.27	72.761±8.832	0,164725468
2	16:0/22:6 DGCC	1.339±0.863	5.346±4.235	0,215579148
3	22:6/22:6 DGCC	1.651±1.224	3.131±3.477	0,183774705
4	22:0/22:6 DGCC	1.446±0.786	2.195±1.83	0,525183939
5	46:8 DGCC	34.257±14.471	16.401±12.028	0,550849723
6	18:0/28:7 DGCC	35.704±15.246	18.596±13.774	0,175776856
7	C_{28} DGCC	59.227±13.27	72.761±8.832	0,222946776

Data are presented as mean ± SD, *n* = 3

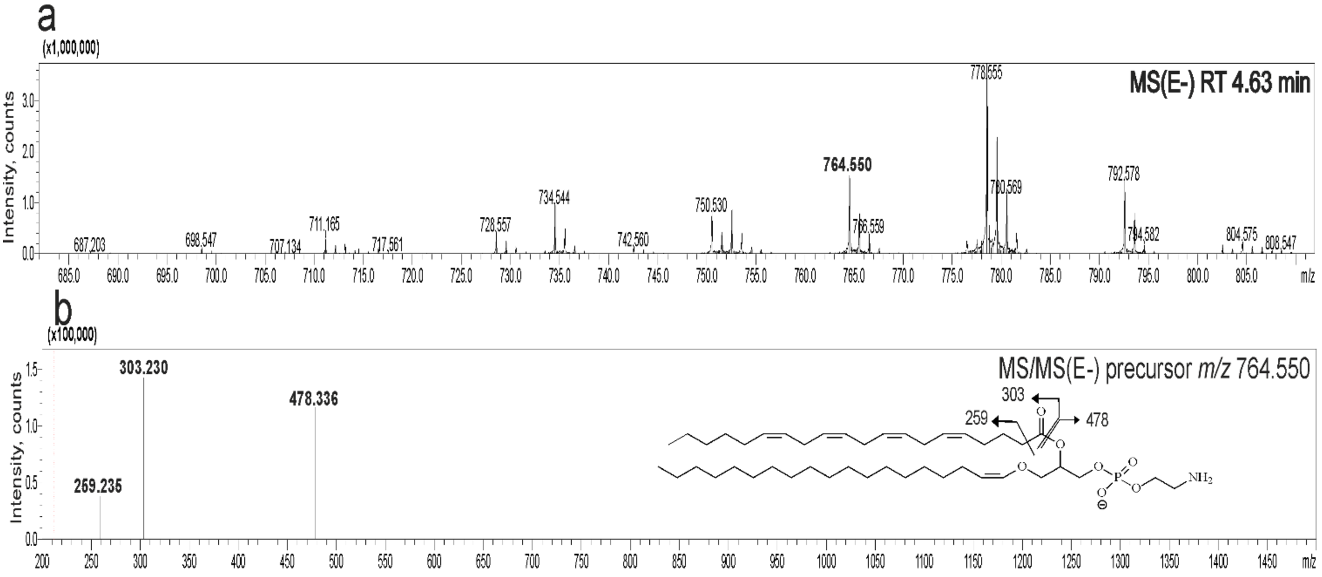

Figure S1 Electrospray ionization mass spectra of proposed 19:1e/20:4 PE. The total lipids of the *J. fragilis* were analyzed by high-performance liquid chromatography with tandem mass spectrometry (HPLC-MS/MS) in negative ion mode. **(a)** Mass spectrum for compounds eluting between 3.7-5.2 min. At tR = 4.63 min, the negative ion $[M–H]^-$ at *m/z* 764.550 was annotated as $[C_{44}H_{80}NO_7P]^-$ (calculated 764.560). **(b)** MS/MS spectrum of the precursor ion at *m/z* 764.550. Diagnostic fragments include the carboxylate anions of 20:4 (*m/z* 303.230) and 19:1 (*m/z* 259.235), the predicted structure of 19:1e/20:4 PE.

Based on the MS/MS fragmentation scheme, this molecular species was identified as *O*-alkyl-acyl-glycerophosphoethanolamine 19:1e/20:4 PE.

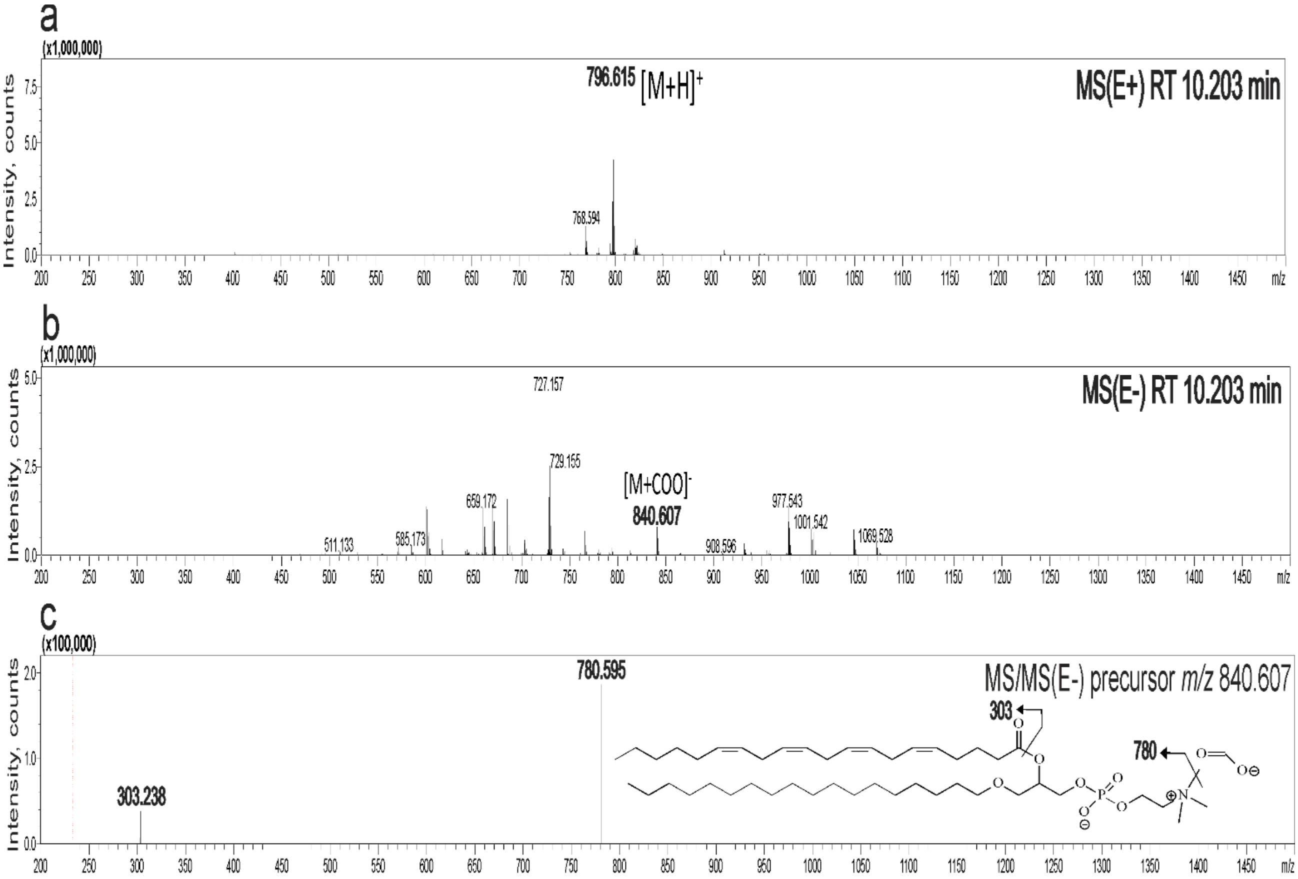

Figure S2 Electrospray ionization mass spectra of proposed 18:0e/20:4 PC. The total lipids of the *J. fragilis* were analyzed by high-performance liquid chromatography with tandem mass spectrometry (HPLC-MS/MS) in negative and positive ion modes. **(a)** Mass spectrum in positive ion mode for compounds eluting between 9.1-11.4 min. At tR = 10.203 min, the positive ion $[M+H]^+$ at *m/z* 796.615 was annotated as $[C_{46}H_{86}NO_7P]^+$ (calculated 796.621). **(b)** Mass spectrum in negative ion mode showing the formate adduct $[M+HCOO]^-$ at *m/z* 840.607 (calculated 840.611). **(c)** MS/MS spectrum of the precursor ion at *m/z* 840.607. The carboxylate anion of 20:4 at *m/z* 303.238 confirms the acyl chain composition, the predicted structure of 18:0e/20:4 PC.

Based on the fragmentation scheme, this molecular species was identified as *O*-alkyl-acyl-glycerophosphocholine 18:0e/20:4 PC.

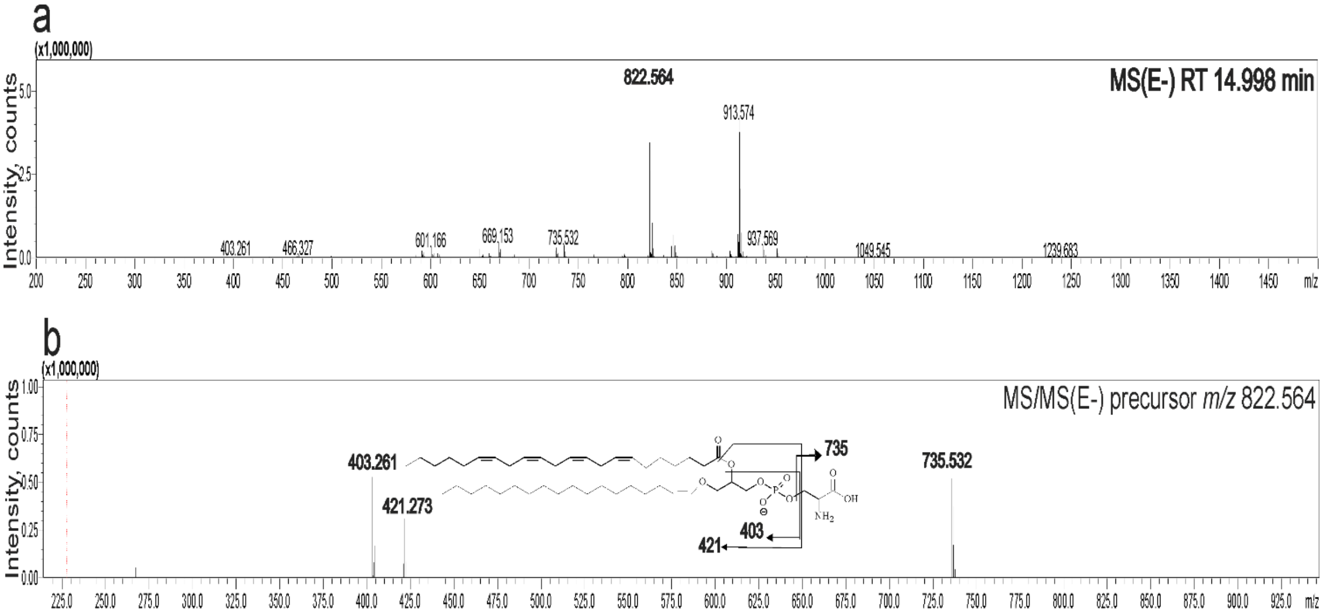

Figure S3 Electrospray ionization mass spectra of proposed 18:1e/22:4 PS. The total lipids of the *J. fragilis* were analyzed by high-performance liquid chromatography with tandem mass spectrometry (HPLC-MS/MS) in negative ion modes. **(a)** Mass spectra for compounds eluting between 13.4-17.9 min. At tR = 14.998 min, the negative ion $[M–H]^-$ at *m/z* 822.564 was annotated as $[C_{46}H_{82}NO_9P]^-$ (calculated 822.565). **(b)** MS/MS spectrum of the precursor ion at *m/z* 822.564. Diagnostic fragments include the loss of 87.032 (serine head group) and fragments at *m/z* 421.273 and *m/z* 403.261, confirming the 22:4 acyl chain, the predicted structures of 18:1e/22:4.

Based on the MS/MS fragmentation scheme, this molecular species was identified as *O*-alkyl-acyl-glycerophosphoserine 18:1e/22:4 PS.

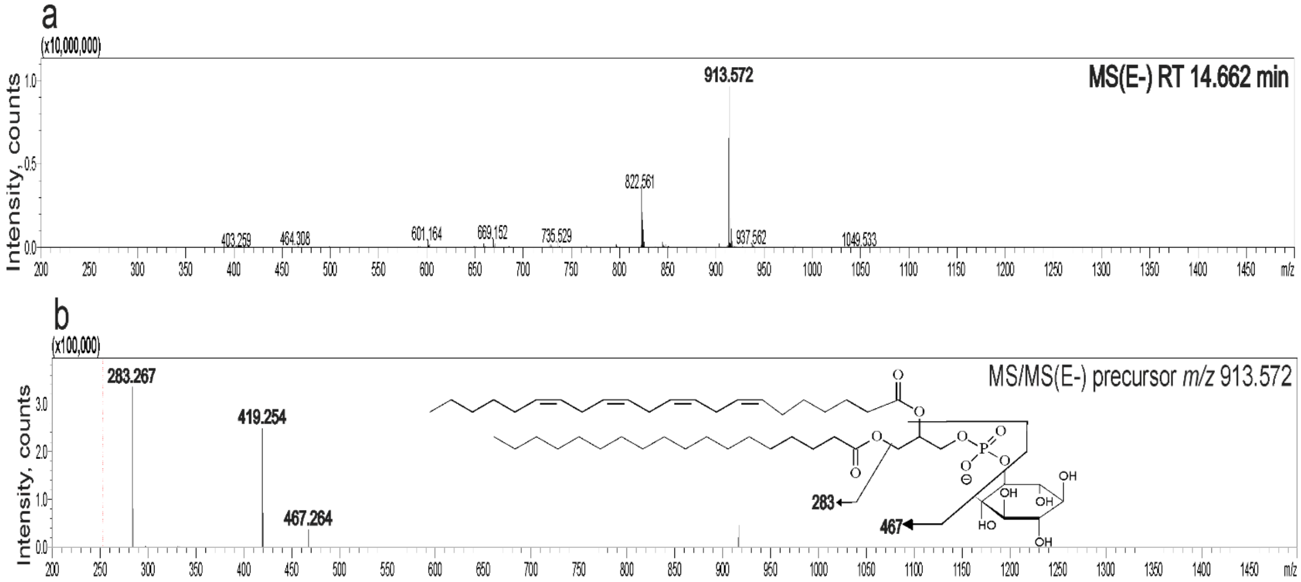

Figure S4 Electrospray ionization mass spectra of proposed 18:0/22:4 PI. The total lipids of the gorgonian corals were analyzed by high-performance liquid chromatography with tandem mass spectrometry (HPLC-MS/MS) in negative ion modes. **(a)** Mass spectrum for compounds eluting between 14.0-15.9 min. At tR = 14.662 min, the negative ion $[M–H]^-$ at *m/z* 913.572 was annotated as $[C_{49}H_{87}O_{13}P]^-$ (calculated 913.581). **(b)** MS/MS spectrum of the precursor ion at at *m/z* 913.572. The carboxylate anion of 18:0 at *m/z* 283.267 and the loss of the inositol head group confirm the structure, the predicted structure of 18:0/22:4.

Based on the MS/MS fragmentation scheme, this molecular species was identified as diacyl-glycerophosphoinositol 18:0/22:4 PI.
